# Cardiac safety in cluster headache patients using the very high dose of verapamil (≥720 mg/day)

**DOI:** 10.1007/s10194-010-0289-x

**Published:** 2011-01-22

**Authors:** M. Lanteri-Minet, F. Silhol, V. Piano, A. Donnet

**Affiliations:** 1 Département d’Evaluation et traitement de la Douleur Médecine palliative, Pôle Neurosciences Cliniques du CHU de Nice, Hôpital Pasteur Avenue de la Voie Romaine, 06002 Nice Cedex, France; 2 INSERM Unité 829, Faculté de Chirurgie dentaire, Clermont-Ferrand, France; 3 Service de Cardiologie, CHU Timone, Rue St Pierre, 13005 Marseille, France; 4 Service de Neurologie, Pôle Neurosciences cliniques, CHU Timone, Rue St Pierre, 13005 Marseille, France

**Keywords:** Cluster headache, Verapamil, Adverse events, EKG monitoring

## Abstract

Use of high doses of verapamil in preventive treatment of cluster headache (CH) is limited by cardiac toxicity. We systematically assess the cardiac safety of the very high dose of verapamil (verapamil VHD) in CH patients. Our work was a study performed in two French headache centers (Marseilles–Nice) from 12/2005 to 12/2008. CH patients treated with verapamil VHD (≥720 mg) were considered with a systematic electrocardiogram (EKG) monitoring. Among 200 CH patients, 29 (14.8%) used verapamil VHD (877 ± 227 mg/day). Incidence of EKG changes was 38% (11/29). Seven (24%) patients presented bradycardia considered as nonserious adverse event (NSAE) and four (14%) patients presented arrhythmia (heart block) considered as serious adverse event (SAE). Patients with EKG changes (1,003 ± 295 mg/day) were taking higher doses than those without EKG changes (800 ± 143 mg/day), but doses were similar in patients with SAE (990 ± 316 mg/day) and those with NSAE (1,011 ± 309 mg/day). Around three-quarters (8/11) of patients presented a delayed-onset cardiac adverse event (delay ≥2 years). Our work confirms the need for systematic EKG monitoring in CH patients treated with verapamil. Such cardiac safety assessment must be continued even for patients using VHD without any adverse event for a long time.

## Introduction

According to quality criteria developed by the American Academy of Neurology [1], verapamil received a grade C rating in a recent meta-analysis of trials of pharmacotherapy for cluster headache (CH) [2]. In spite of this low evidence level, verapamil is generally considered to be the mainstay of CH preventive therapy as in the European guidelines [3]. The starting daily dose of verapamil in CH should be the 360 mg effective in two randomized clinical trials [4, 5]. The daily dose could be increased up to 720 mg and some CH patients may even need unusual very high daily dose from 720 to 1,200 mg [6]. Considering such a clinical practice, the dose of verapamil used for CH is approximately twice the dose required by cardiovascular diseases [7]. This difference could be explained by the fact that the cardiovascular effects are related to blood level, whereas the preventive CH effect takes place across the blood–brain barrier where the efflux transporter P-glycoprotein restricts net brain uptake of verapamil by immediately transporting it out of the brain [8]. Considering the use of high doses, the cardiac safety of verapamil therapy was specifically studied in one series that included 108 CH patients treated by verapamil with systematic electrocardiogram (EKG) assessment [9]. In this series, verapamil was started at 240 mg/day and then increased until the CH was suppressed, or to a maximum daily dose of 960 mg (mean daily dose 584 ± 257 mg) and incidence of arrhythmia was 19% and bradycardia 36% [9]. We developed a similar approach to assess the cardiac safety of verapamil therapy in CH with a focus on very high daily dose equal or higher than 720 mg/day.

## Methods

The notes were assessed for patients with episodic CH or chronic CH attending two headache specialty centers (Marseilles and Nice) belonging to the French Observatory of Migraine and Headaches [10] from December 2005 to December 2008. Patients had a diagnosis of CH according to the criteria of the second edition of the International Classification of Headache Disorders [11]. When the verapamil was used, the starting dose was 360 mg with an increase by 120 mg every 2 weeks with a check EKG, until the CH was suppressed or adverse events intervened. Ordinary release formulation or controlled release formulation were both used.

Study considered CH patients using verapamil with a very high daily dose defined as ≥720 mg. The following data were collected for each patient: sex, age, tobacco use and cardiovascular history, diagnosis (episodic or chronic CH), duration of verapamil use, very high dose of verapamil achieved, duration of use of such a very high dose of verapamil, concomitant medications, clinical adverse events related to verapamil (constipation, lethargy, hypotension, lower edema, dyspnea, impotence, gingival hyperplasia). EKG assessment before verapamil introduction was compared with EKG assessment done at the very high dose of verapamil achieved.

## Results

### Patients

Among 200 CH identified seen during the study period, 29 (14.8%) used verapamil with a daily dose ≥720 mg. Very high verapamil dose CH patients were 28 men and 1 woman with a mean age 43.2 ± 10 years (range 21–55 years). Twenty one were present smokers, six were past smokers and two had never smoked. Three had a high blood pressure and one a coronary heart disease. Nine suffered of episodic CH and 20 of chronic CH. Mean duration of verapamil therapy was 46 ± 36 months and mean duration of very high dose use was 36 ± 32 months. Mean very high dose of verapamil was 877 ± 227 mg/day (720 mg: 16; 840 mg: 2; 960 mg: 7; 1,200 mg: 1; 1,440 mg: 3). Concomitant treatments for CH (acute and prophylactic) are presented in Table 1.

**Table 1:** Cardiac safety of the very high verapamil CH patients

	Sex	Age	TU	CVH	CH	vD	vSMD	SM	ACT	PCT	EKG changes	SAE	vC	CAE
1	M	50	Present	No	C	108	96	1,200	scS	No	No			No
**2**	**M**	**21**	**No**	**No**	**E**	**84**	**72**	**960**	**scS**	**No**	**Second degree HB**	**Yes**	**Stop**	**No**
*3*	*M*	*45*	*Present*	*No*	*E*	*12*	*2*	*960*	*scS*	*No*	*Bradycardia*	*No*	*No*	*No*
*4*	*M*	*47*	*Present*	*No*	*C*	*72*	*60*	*840*	*scS*	*T*	*Bradycardia*	*No*	*No*	*No*
5	M	55	Past	HBP	E	1	1	720	scS	No	No			D
6	M	34	Present	No	C	12	1	720	scS	No	No			No
*7*	*M*	*50*	*Present*	*No*	*C*	*36*	*24*	*1,440*	*scC*	*No*	*Bradycardia*	*No*	*No*	*L*
8	M	33	Present	No	E	48	1	720	scC	No	No			No
9	M	46	Past	No	E	1	1	720	scC	No	No			L
10	M	37	Present	No	E	1	1	720	scC	No	No			C
11	M	72	Present	No	E	3	2.5	960	O_2_	No	No			C
**12**	**M**	**51**	**Past**	**No**	**C**	**36**	**24**	**1,440**	**oZ**	**No**	**Third degree HB**	**Yes**	**Stop**	**L–D**
13	M	49	Present	No	C	120	96	960	scC	L	No			No
**14**	**M**	**52**	**Present**	**No**	**E**	**1**	**1**	**840**	**scC**	**No**	**First HB**	**Yes**	**↓600**	**D–E**
*15*	*M*	*30*	*No*	*No*	*E*	*1*	*1*	*960*	*No*	*No*	*Bradycardia*	*No*	*No*	*No*
*16*	*M*	*33*	*Present*	*No*	*C*	*25*	*24*	*720*	*scS*	*T-I*	*Bradycardia*	*No*	*No*	*L*
**17**	**M**	**37**	**Present**	**HBP**	**C**	**72**	**71**	**720**	**scC**	**G**	**First HB**	**Yes**	**↓840**	**No**
18	M	40	Present	No	C	47	46	960	scC	No	No			L–E
19	M	53	Past	HBP	C	96	93	720	scC	No	No			L–D–I
20	M	37	Present	No	C	76	53	720	scC-O_2_	No	No			L–D–E
21	M	42	Present	No	C	17	10	720	scC	I	No			L–E–G
22	W	34	Past	No	C	18	10	720	scC-O_2_	No	No			L
23	M	55	Present	No	C	53	51	720	scC	No	No			L
24	M	41	Present	No	C	84	58	960	scC	No	No			L–D–E
*25*	*M*	*28*	*Present*	*No*	*C*	*37*	*36*	*720*	*scC-O*_*2*_	*No*	*Bradycardia*	*No*	*No*	*L*
26	M	31	Present	No	C	81	46	720	scC	I	No			No
*27*	*M*	*52*	*Present*	*No*	*C*	*27*	*24*	*1,440*	*scC*	*I*	*Bradycardia*	*No*	*↓1,200*	*No*
28	H	54	Past	CAD	C	71	53	720	scC	No	No			No
29	H	33	Present	No	C	96	85	720	scC	No	No			L

Sex, age (years), tobacco use (TU), cardiovascular history (CVH) with high blood pressure (HBP) and coronary arteries disease (CAD), type of CH (E: episodic CH–C: chronic CH), duration of verapamil use (vD) in months, duration of supra-maximum dose of verapamil (vSMD) in months, supra-maximum dose of verapamil achieved (SM) in mg/day, acute concomitant treatments (ACT/scC: subcutaneous sumatriptan–oZ: oral –O_2_: oxygen), prophylactic concomitant treatments (PCT/I: indomethacin–G: gabapentin–L: lithium–T: topiramate), electrocardiogram (EKG) changes, serious adverse event (SAE), change in verapamil dose (vC), clinical adverse events (CAE/C: constipation–D: dyspnea–E: edema of lower limbs–G: gingival hyperplasia–I: impotence–L: lethargy)

Patients with serious cardiac adverse event are in bold and patient with nonserious cardiac adverse event are in italics

### EKG changes

EKG changes concerned 11 (38%) patients: bradycardia (heart rate <60 bpm) in 7 patients, first-degree heart block (PR interval >0.2 s) in 2 patients, second-degree heart block in 1 patient and third degree heart block in 1 patient. Patients with EKG changes used a mean verapamil daily dose of 1,003 ± 295 mg, whereas patients without EKG changes used a mean verapamil dose of 800 ± 143 mg. EKG changes have been considered as cardiac serious adverse event (SAE) in the four (14%) patients with heart block inducing verapamil discontinuation in two patients and a dose reduction in one patient. EKG changes have been considered as cardiac nonserious adverse event (NSAE) in seven (24%) patients with bradycardia, but verapamil dose was decreased in one patient.

Cardiac SAE concerned 4 men with mean age 40.2 ± 14.5 years (range 21–52 years) and using a mean very high verapamil daily dose of 990 ± 315 mg. One patient had a high blood pressure history and regarding tobacco use, two were present smokers, one was past smoker and one patient had never smoked. Cardiac SAE concerned patients using verapamil without concomitant medications expect sumatriptan or zolmitriptan as acute treatment. Cardiac SAE were delayed onset in three patients (72, 71 and 24 months after the very high dose was achieved). Cardiac SAE were asymptomatic in two patients and symptomatic in two patients with lethargy, and dyspnea for one patient and lethargy for the other.

Cardiac NSAE concerned seven men with mean age 40.7 ± 10 years (range 28–52 years) and using a mean very high verapamil daily dose of 1,011 ± 309 mg. No patient had cardiovascular history and regarding tobacco use, six were present smokers and one had never smoked. Cardiac NSAE concerned one patient without any concomitant treatment, three patients without concomitant treatment except acute treatment (sumatriptan and oxygen) and three patients with prophylactic concomitant treatment (topiramate and/or indomethacin). Cardiac NSAE was delayed onset in five patients (60, 36, 27, 24 and 24 months after the very high dose was achieved). Cardiac NSAE were asympomatic in four patients and associated lethargy in the three other patients.

## Discussion

Considering the frequent use of high daily doses, cardiac safety assessment with systematic EKG monitoring is essential in the management of CH patients treated by verapamil [7]. This is all the more essential as the very high daily dose (≥720 mg) is used. The use of the very high daily dose is not infrequent and our study showed that it corresponds to 14.8% of CH patients managed in two centers representative of French headache tertiary centers. In this group patients treated with the very high daily dose of verapamil (877 ± 227 mg), systematic EKG monitoring demonstrated that incidence of cardiac adverse events is 38%, with more than one-third of cases, the occurrence of an adverse event was considered as serious. Cardiac SAE were arrhythmias induced by reduction of transmission in the atrioventricular node: first-degree heart block in two patients needing daily dose reduction, second- and third-degree heart block in two others patients needing verapamil discontinuation. In a previous study on 108 CH patients using a mean daily dose of 584 ± 264 mg, incidence of arrhythmia (mostly first-degree heart block and junctional rhythm) was 19% and bradycardia 36% [9]. In this study, patients with arrhythmia (567 ± 290 mg/day) were not taking higher doses than those without arrhythmia (586 ± mg/day) [9]. By contrast, we found that patients with EKG changes (1,003 ± 295 mg/day) were taking higher doses than those without EKG changes (800 ± 143 mg/day), but doses were similar in patients with cardiac SAE (990 ± 316 mg/day) and those with cardiac NSAE (1,011 ± 309 mg/day). In our study, cardiac adverse events were not related to the patients’ age, cardiovascular history, CH type and concomitant drugs used for acute and/or prophylactic treatment of CH. All these data are congruent with those previously reported [9] and could be related to an interindividual variability in the pharmacology of verapamil supported by a genetic component [7]. This hypothesis was developed in the cardiologic field and data collected in the INVEST suggests variability in the large-conductance and voltage-dependant potassium channel beta 1 subunit gene, KCNMB1, is associated with the antihypertensive response to verapamil and also with cardiovascular adverse events in patients having hypertension with coronary arteries disease [12]. Our study also suggests an important intra-individual variability in the risk of cardiac adverse events. Such an intra-individual variability could explain the delayed onset of cardiac adverse events which is probably the more striking data collected in our study. Late-onset arrhythmia was previously described in two CH patient treated with verapamil [9]. Around three-quarters (8/11) of our patients presented cardiac adverse events with a delayed onset, this proportion being similar to bradycardia (5/7) and arrhythmia (3/4). In all these late-onset adverse events cases, the time between the adverse event occurrence date and that corresponding to the very high daily dose verapamil use was equal or higher than 2 years. This confirms the need of regular and systematic EKG monitoring as EKG abnormalities can develop insidiously with rising subthreshold PR intervals, or suddenly after long time of normal EKGs. This systematic assessment is all the more important than cardiac adverse events can occur without any other clinical adverse event as in four of our eight cases with late-onset cardiac adverse event.
